# Supported to perform: sports bras and breast volume do not impair cycling performance in females

**DOI:** 10.3389/fspor.2024.1439403

**Published:** 2024-10-11

**Authors:** Camilla R. Illidi, Dennis Jensen

**Affiliations:** ^1^ Clinical Exercise and Respiratory Physiology Laboratory, Department of Kinesiology and Physical Education, McGill University, Montréal, QC, Canada; ^2^ Sylvan Adams Sports Science Institute, Faculty of Education, McGill University, Montréal, QC, Canada; ^3^ Translation Research in Respiratory Diseases Program, Research Institute of the McGill University Health Centre, Montréal, QC, Canada

**Keywords:** exercise performance, cycling, female anthropometry, sports brassiere, breathlessness

## Abstract

**Introduction:**

Despite the importance of sports bras for comfort during exercise in people with breasts, concerns persist regarding their potential effects on athletic performance. Discrepancies in previous studies necessitate a closer examination of the interaction between sports bras, breast volume, exertional symptoms, and exercise performance.

**Methods:**

Twenty-three recreationally-active, normal bodyweight females completed three 10-km time-trials on a cycle ergometer on three separate occasions in a randomized order, while wearing a professionally fitted high-support sports bra, a professionally fitted low-support sports bralette, or a personal, self-selected sports bra. Performance was quantified as the time to complete the 10-km distance. Cardiorespiratory and symptom responses were measured throughout.

**Results:**

Participants were grouped by their estimated breast volumes (small: mean ± SD 284 ± 38 ml, median bra size: 32C; large: 560 ± 97 ml, 34DD; *p* = 0.002, *g* = 3.84). The average time-trial duration was 23.1 ± 3.1 min and comparable across breast volume groups and sports bra conditions (between-group: *p* = 0.794, *η*_p_^2^ < 0.01; between-bras: *p* = 0.273, *η*_p_^2^ < 0.01). Notably, larger-breasted participants experienced stronger symptoms of chest tightness (*p* = 0.042, *η*_p_^2^ = 0.18), which were associated with their ratings of perceived exertion and breathlessness (intensity and unpleasantness). Irrespective of breast volume, the high-support sports bra also evoked stronger symptoms of chest tightness (*p* = 0.039, *η*_p_^2^ = 0.15).

**Discussion:**

Stronger symptoms of chest tightness associated with larger breast volumes or high-support sports bras do not impede performance during self-paced non-weight-bearing exercise in recreationally-active females.

## Introduction

Studies exploring the effects of sports bras and breast volume on exercise performance and its psycho-physiological determinants has shown varying results. For instance, Bowles et al. (1) found no effect of bra type (fashion bra, compression bra, or encapsulation bra) on the rate of oxygen uptake (V̇O_2_) during submaximal and maximal exercise, and negligible effects of breast volume on breathing patterns during exercise. By contrast, a study in highly trained female endurance athletes found that V̇O_2_, minute ventilation (V̇_E_) and the work of breathing were higher during treadmill running while wearing sports bras with tight compared to loose underbands (2). We recently reported no effects of breast volume or optimally fitted sports bras of various support levels on breathing mechanics, respiratory muscle pressure development or inspiratory neural drive (diaphragm EMG) during non-weight-bearing cycle ergometer exercise (3). Accordingly, there is little evidence that breast volume or professionally-fitted fitted sports bras adversely affect exercise performance via clear respiratory physiological mechanisms.

However, exercise performance can also be affected by negative sensory experiences. For instance, dyspnea (breathlessness) can cause negative emotions and consequently reduce exercise performance in otherwise healthy individuals (4–6). We recently found that females with larger breast volumes (LBV; bra sizes ≥34DD) systematically reported higher ratings of perceived exertion (RPE) and breathlessness intensity and unpleasantness compared to females with smaller breast volumes (SBV; bra sizes <34DD) at similar absolute and relative exercise intensities on a stationary cycle ergometer (3). We also found that these heightened symptoms in LBV participants were associated with higher ratings of perceived chest tightness due to their bra, particularly when wearing high-support sports bras (3). It follows that during self-paced exercise (e.g., time-trial), LBV people may exercise at a lower power output (exercise intensity) than their SBV counterparts to maintain their exertional symptoms within tolerable limits, but at the expense of relatively worse performance. However, no study has empirically tested this hypothesis. Therefore, this study assessed the effects of optimally fitted sports bras and breast volume on 10-km cycling time-trial performance. We purposefully selected non-weight-bearing stationary bicycle exercise to minimize the influence of breast motion, body mass index (BMI), and body mass on our performance outcomes. We hypothesized that LBV compared to SBV participants would exercise at a lower exercise intensity (power output) to maintain similar ratings of exertional symptoms, ultimately leading to inferior 10-km cycling time-trial performance. We further hypothesized that these between-group differences in performance would be exaggerated when wearing a high- compared to a low-support sports bra.

## Methods

### Ethical approval

The study was approved by the Institutional Review Board of the Faculty of Medicine and Health Sciences at McGill University (A11-M78-22A). Written informed consent was obtained from all participants after a detailed written and verbal explanation of the experimental protocol. The study conformed to the standards set by the Declaration of Helsinki, except for registration in a database.

### Experimental overview

**Figure 1:**
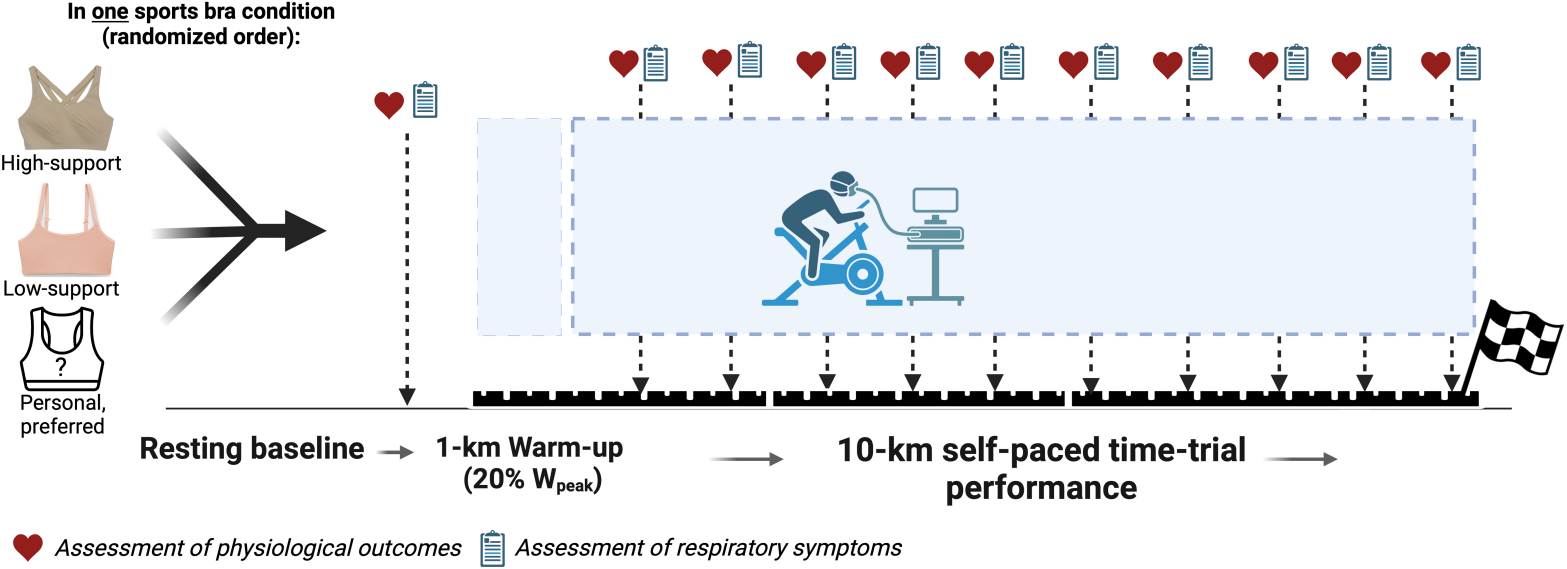
Schematic of the study protocol, with the assessments of physiological and symptom outcomes at the end of resting baseline and every 750 m of each kilometer. Created with BioRender.com.

The study consisted of four laboratory visits to the Clinical Exercise & Respiratory Physiology Laboratory at McGill University. The first visit was for screening, assessment of baseline participant characteristics, and familiarization with the experimental protocols. While wearing their personal sports bras (see details below), participants also completed a symptom-limited incremental cardiopulmonary cycle exercise test (CPET) to determine peak power output (W_peak_) and peak rate of oxygen consumption (V̇O_2peak_). As shown in Figure 1, the subsequent three experimental visits each included a 10-km cycling time-trial and were randomized to: (i) a high-support compression sports bra; (ii) a low-support sports bralette; or (iii) the participants’ preferred personal bra, irrespective of brand, size, design, or support (control). Each participant's breast volume was estimated from their bra size, as described by McGhee & Steele (7), and used for group allocation to a small breast volume (SBV) or large breast volume (LBV) group according to the median estimated breast volume.

For participants who self-reported not using hormonal contraceptives and/or cyclic mastalgia (breast pain), experimental visits were scheduled around the luteal phase to avoid the confounding influence of breast sensitivity on symptom responses to exercise under each sports bra condition (8). All experimental visits were separated by 2–5 days and scheduled for the same time of day for each participant. Participants were asked to avoid strenuous exercise for 24 h. prior to each visit, and alcohol and caffeine on the day of each visit.

### Participants

Twenty-three healthy, recreationally-active females aged 18–27 years participated in this study. Participants met the World Health Organization's recommendations for self-reported physical activity of ≥150 min/week of moderate-intensity activity or ≥75 min/week of vigorous-intensity activity (9), and self-reported no chronic health conditions, no doctor-prescribed medication use (except hormonal contraceptives), and no contraindications to exercise. Inclusion criteria were body mass index (BMI) of 18.5–24.9 kg/m^2^; dual-energy x-ray absorptiometry (DXA)-derived whole-body fat mass (FMI, kg/m^2^) and lean mass indices (LMI, kg/m^2^) within 25th–75th percentiles of the sex, age and BMI-specific normal range (10); and ratio of forced expiratory volume in 1 s to forced vital capacity (FEV_1_/FVC)≥0.70.

### Questionnaires and menstrual history

To determine eligibility, participants completed a medical history form and the Physical Activity Readiness Questionnaire for Everyone (PAR-Q+) and answered questions about their physical activity habits. If entered into the study, participants were further asked questions pertaining to their menstrual history to self-identify hormonal contraceptive use, premenstrual symptoms (including cyclic mastalgia), and the duration of menstrual cycle phases to schedule timing of experimental visits, as per the recommendations of Elliot-Sale et al. (11).

### Spirometry

Pulmonary function was assessed via spirometry using automated testing equipment (Vmax Encore, Trudell Healthcare Solutions, London, ON, Canada) in accordance with recommended standards (12) and variables referenced to race-neutral prediction values (13).

### Dual-energy x-ray absorptiometry (DXA)

Whole-body composition was assessed using a Lunar iDXA scanner (GE Healthcare, Milwaukee, US) and dedicated software for subsequent analysis (GE Lunar Encore, v.13.60). In addition to FMI and LMI (see above), values obtained included whole-body fat mass (FM), lean mass (LM), and fat percentage. We also estimated tissue distribution and mass within the upper thorax (an estimate of total breast mass) by creating a customized region of interest, covering the surface from the clavicles to the xiphoid process, between the left and right anterior axillary lines (see Supplementary Figure 2).

### Bra fitting and sports bra conditions

To determine the correct bra size for our experimental sports bras, we first used fashion bras from a manufacturer whose sizing chart was identical to that of the sports bras used in the study. The underband size was identified by measuring chest circumference with a tape measure and cross-referencing this with the manufacturer's sizing guide. We further ensured that the underband was fitted horizontally across the back while still being tight enough to be lifted ≤1 in. from the back. The cup size was determined through visual assessment and fit criteria, including fabric tension, wire alignment, and overall coverage. The straps were adjusted to fit snugly, allowing one finger's width between the strap and the shoulder, with even pressure to allow for smooth movement of the finger across the strap. After the correct size had been identified, we confirmed that our experimental sports bras followed the same criteria as described above.

For our experimental sports bras, the high-support condition used a compression bra (78% nylon, 22% elastane) with double-crossback straps for an even distribution of support and compression (lululemon Energy High Support, lululemon athletica inc., Vancouver, British Columbia, Canada). The low-support condition used a wireless sports bralette (79% nylon, 21% elastane) with contour pads and conventional, vertical straps (Natori Limitless, The Natori Company, New York City, US). For our control condition (preferred, personal sports bra), participants were asked to bring their favorite sports bra for high-intensity exercise, without any restrictions on size, fit, design, support, color, or brand. The intention was to assess performance outcomes in a sports bra that participants were sure to be comfortable in, despite the possibility that its size, support, and fit might be suboptimal. The participants’ personal sports bras spanned a wide range of designs, including bandeau bras, plunge bras, and low-support compression bras (see Supplementary Figure 1 for bra conditions, including examples of personal sports bras). Due to frequent wear, the specific sizes of most personal bras could not be determined; however, all were models using either numerical (e.g., 6, 8, 10) or alpha sizing (e.g., S, M, L). Only one participant (SBV) reported having undergone a professional bra fit in the past, but her personal sports bra still used numerical sizing. As such, none of the participants brought sports bras that had been professionally fitted to their size or sporting needs.

### Cardiopulmonary cycle exercise testing and time-trial

All exercise tests were conducted on an electronically braked upright cycle ergometer (Velotron Pro with Version 1.6 of the Velotron Coaching Software, RacerMate Inc., Seattle, WA, UA) using a Vmax Encore® metabolic cart (Trudell Healthcare Solutions, London, ON, Canada). Gas exchange, V̇_E_, tidal volume (V_T_) and respiratory frequency (*f*_R_) were collected breath-by-breath at rest prior to the start of exercise (resting baseline) and during exercise while participants breathed through a low-resistance flow transducer and silicone facemask (Hans Rudolph, 7450 Series V2, Shawnee, KS, US). Heart rate (HR) was monitored continuously using a Polar H10 heart rate monitor (Polar Electro Oy, Kempele, Finland).

Incremental CPETs consisted of a 3-min baseline period, followed by 25-W/min increments in power output, starting at 25 W. Participants were verbally encouraged to maintain a pedal cadence of 70–90 rev/min and to exercise to the point of volitional fatigue (symptom limitation) or until they were unable to maintain a pedal cadence >70 rev/min for >5 s. Peak work rate (W_peak_) was defined as the highest power output the participant was able to sustain for ≥30-s, whereas the V̇O_2_ and V̇_E_ at peak exercise were taken as the V̇O_2_ and V̇_E_ averaged over the last 30-s of loaded pedaling (V̇O_2peak_ and V̇_Epeak_, respectively). Each participant's W_peak_ and V̇O_2peak_ responses were compared to their respective prediction values from the American College of Sports Medicine (14) and reference standards by Kaminsky et al. (15) to ensure that all participants reached V̇O_2peak_ at or above the 50th percentile of their age- and sex-specific reference group. To assess the individual and combined effect of sports bra support and breast volume on exercise performance, following a pre-exercise rest period of 3 min and a 1 km warm-up at 20% of W_peak_, participants performed a 10 km cycling time trial. Participants were instructed to perform the time-trials “as fast as possible” by maintaining the highest cycling speed possible (see Figure 1 for schematic of study outline). Participants were blinded to their time-trial duration and power output but received real-time visual feedback on their pedal cadence, distance, and cycling gear. Participants were not provided verbal feedback, encouragement and/or instructions during the 10-km cycling time trials.

### Symptom responses

As shown in Figure 1, at rest prior to exercise and within the final 250 m of every kilometer interval during each 10-km time-trial, participants rated each of the following symptoms using Borg's modified 0–10 category ratio scale (16): RPE (“How intense is your sensation of effort overall?”); breathlessness intensity (“How intense is your sensation of breathing overall?”); breathlessness unpleasantness (“How unpleasant or bad does your breathing make you feel?”); and intensity of their perceived chest tightness due to their bra (“How tight or restrictive is your bra around your rib cage?”). Prior to exercise at each visit, participants were familiarized with the Borg CR10 scale and its endpoints were anchored such that ″0″ represents “no intensity (unpleasantness) at all” and ″10″ represents “the most severe intensity (unpleasantness) you have ever experienced or could ever imagine experiencing.”

### Bra tightness and breast acceleration

Bra underband tightness was measured breath-by-breath during the pre-exercise baseline period using the PowerLab data-acquisition system and calibrated differential pressure transducers (model DP15-34, Validyne Engineering, Northridge, CA) by affixing two balloon-tipped pressure catheters (Ackrad Laboratories, Cranford, NJ, USA), each filled with 2 ml of air and taped with surgical tape on the non-inflatable part of the balloon beneath the underband of the bra: directly below the left nipple (anterior) and at the left mid-axillary line (lateral) (see Supplementary Figure 3).

Breast acceleration was quantified in anteroposterior, mediolateral, and superior-inferior directions using inertial measurement units (XSens DOT, Movella Inc., Henderson, NV, US), with one sensor taped to the sternum and one placed inside the sports bra on the right nipple. Raw acceleration data were recorded at 60 Hz using a commercially available data acquisition application (v2020.4.1, Movella Inc.) and analyzed with a bespoke MATLAB code designed for this purpose. The vector magnitude of breast acceleration, which combines the acceleration components in all three directions, was calculated and reported in cm/s² to provide a measure of overall acceleration. The raw data were filtered with a 20 Hz low-pass filter and averaged over the entire duration of the 10-km time-trial.

### Analysis of exercise end-points

Physiological variables measured breath-by-breath during experimental visits were averaged over (i) the last 60-s of the 3-min pre-exercise baseline period and (ii) the last 30-s of each 1-km interval of the 10-km time-trial. These variables were then linked with contemporaneous measures of HR, RPE, breathlessness intensity and unpleasantness, and ratings of perceived chest tightness due to bra. To account for differences in peak exercise capacity, pulmonary function, and body composition between SBV and LBV participants, V̇_E_ was normalized to V̇_Epeak_ (V̇_E_%peak), V_T_ was normalized to FVC (V_T_%FVC), and both power output and V̇O_2_ (V̇O_2LM_) were normalized to DXA-derived measures of whole-body LM.

#### Sample size estimation

Using a two-way (bra condition × breast size) mixed-effects analysis of variance (ANOVA) with α = 0.05 and a statistical power of 0.90, at least eight participants were needed in each group to detect a minimal difference of 5% in 10-km time-trial duration between sports bras and/or breast volumes. This difference in 10-km time-trial duration is twice the within-athlete variation in 10-km duration typically seen in recreationally-active adults (17).

#### Statistical analyses

Statistical analyses were performed using dedicated software (SPSS Statistics, v.29, IBM Corp., Armonk, NY; GraphPad Prism, v.10, GraphPad Software, Boston, MA). Variables were assessed for normal distribution and, if violated, log transformed (Log10). Participant demographics were expressed as mean ± SD for the total sample and separately for SBV and LBV groups. Between-group (SBV, LBV) differences in baseline characteristics were compared using two-tailed, independent samples *t*-tests, with the magnitudes of reported effects expressed as Hedges *g*. Effect sizes were interpreted as small (*g* = 0.20–0.49), medium (*g* = 0.50–0.79), or large (*g* ≥ 0.80) (18).

A three-step approach guided our statistical methods for the exercise data. First, we examined the main effects of sports bra condition (high-support, low-support, personal) and breast volume (SBV, LBV) on time-trial duration with a mixed-model ANOVA. Second, we examined the same effects (sports bra condition, breast volume) on power output (absolute and relative to LM) and V̇O_2_ (absolute V̇O_2_ and V̇O_2LM_) for each kilometer of the time-trial. These two steps allowed us to (i) determine if there was an effect of sports bra and/or breast volume on our primary performance outcome (time-trial duration) and (ii) whether participants exercised at [dis]similar intensities despite potentially completing the time-trials in similar durations.

As there was no significant effect of sports bra and/or breast volume at any kilometer of the 10-km time-trials, we proceeded to average all outcomes across each kilometer for the three time-trials. Using these averaged data, we finally performed a mixed-effects ANOVA to determine the main effects of sports bra support and breast volume on power output, cardiorespiratory outcomes (V̇_E_, V_T_, *f*_R_, HR, respiratory quotient [RQ], V̇O_2LM_), and symptom responses (RPE, breathlessness intensity, breathlessness unpleasantness, and chest tightness due to bra). For within-subject factors (sports bra condition), sphericity was assessed with Mauchly's test, and if violated, a Greenhouse-Geisser adjustment was used. Due to the unequal sample size in the SBV and LBV groups and mixed-factors study design, all ANOVAs were performed without applying corrections for multiple comparisons before we adjusted all *p*-values for the false discovery rate associated with multiple comparisons. The magnitudes of reported effects from ANOVA are expressed as partial eta squared (*η*_p_^2^) and interpreted as small $\left(\eta_{p}^{2}\;=\;\;0.01-0.05\right)$, medium $\left(\eta_{p}^{2}\;=\;\;0.06-0.13\right)$ or large $\left(\eta_{p}^{2}\;\geq \;0.14\right)$ (18).

Finally, the associations between symptom responses (RPE, breathlessness intensity and unpleasantness, chest tightness due to bra) were quantified with Pearson's correlation coefficients (r) and compared between groups (SBV, LBV) using Fisher's *r*-to-*z* transformation and a two-tailed z-test for independent samples (19). All data are expressed as mean ± SD unless stated otherwise. The alpha level was set as 0.05.

## Results

### Participants

As intended, SBV and LBV participants measured different breast volumes and thoracic tissue distribution (Table 1). The median bra size of SBV participants was 32C (range 32B to 34D), whereas LBV participants were fitted to a median bra size of 34DD (range 34DD to 36DDD). Compared to SBV participants, LBV participants had greater body mass (64.5 ± 2.9 kg vs. 68.2 ± 6.0; *p* = 0.016, *g* = 0.75) and whole-body FM (16.2 ± 2.5 kg vs. 19.4 ± 2.3 kg; *p* = 0.019, *g* = 1.28). The differences in whole-body FM were partially attributed to differences in thoracic FM, whereby LBV participants carried ∼1.1 kg more FM on their thoraces than SBV participants (see Table 1). The two groups were of similar height, age, and whole-body LM (all *p* ≥ 0.05). The two groups also exhibited similar pulmonary function (all *p* > 0.05): FVC 4.56 ± 0.72 L (117 ± 12% predicted); FEV_1_ 3.69 ± 0.50 L (110 ± 10% predicted); FEV_1_/FVC 0.82 ± 0.02 (94 ± 8% predicted).

**Table 1:** Breast and thoracic body composition metrics in small-breast volume (SBV) and large-breast volume (LBV) participants with associated *p*-values and effect sizes (*g*) for between-group comparisons.

	SBV (*n* = 12)	LBV (*n* = 11)	*P*-value (SBV vs. LBV)	Effect size (*g*)
Underband size^a^	32 (32–34)	34 (32–36)	–	–
Bra cup size^a^	C (B–C)	DD (B–DDD)	–	–
Estimated breast volume (ml)	284 ± 38	560 ± 97	**0.002**	**3.84**
Thoracic fat mass (kg)	1.51 ± 0.39	2.60 ± 0.63	**0.001**	**2.12**
Thoracic fat mass (% whole-body fat mass)	9.3 ± 2.1	13.4 ± 2.5	**0.003**	**1.79**

*P*-values and effect sizes highlighted in bold are statistically significant (*P* < 0.05).

^a^ Reported as median (min-max range).

Exercise responses from incremental CPET also showed that the two groups reached similar peak exercise responses (all *p* > 0.05, see Table 2). Self-reported training volume was also similar between SBV and LBV participants (SBV: 8.9 ± 3.5 h/week vs. LBV: 9.3 ± 6.8 h/week; *p* = 0.871, *g* = 0.07) and included walking for transportation and recreation, jogging, swimming, cycling, resistance training, and ball games. Two participants (one SBV, one LBV participant) were former high-level swimmers but had been exercising recreationally for ≥3 months.

**Table 2:** Peak responses from incremental, symptom-limited cardiopulmonary cycle exercise testing (CPET) in small-breast volume (SBV) and large-breast volume (LBV) participants with associated *p*-values and effect sizes (*g*) for between-group comparisons.

	SBV (*n* = 12)	LBV (*n* = 11)	*P*-value (SBV vs. LBV)	Effect size (*g*)
V̇O_2_peak (L/min)	2.66 ± 0.33	2.93 ± 0.68	0.934	0.03
V̇O_2_peak (ml/min/kg BM)	43.3 ± 6.7	43.4 ± 6.9	0.986	0.02
V̇O_2_peak (ml/min/kg LM)	64.2 ± 9.6	61.5 ± 8.4	0.485	0.29
V̇O_2_peak (% predicted)	108 ± 11	116 ±7	0.051	0.87
Wpeak (Watts)	221 ± 30	229 ± 43	0.576	0.22
Wpeak (Watts/kg·BM)	3.56 ± 0.56	3.46 ± 0.75	0.704	0.15
Wpeak (Watts/kg·LM)	5.33 ± 0.81	5.01 ± 0.71	0.328	0.42
Wpeak (% predicted)	106 ± 15	104 ± 16	0.760	0.13

V̇O_2_ peak, rate of O_2_ consumption at the symptom-limited peak of incremental CPET; BM, whole-body mass; LM, whole-body lean mass; Wpeak, power output at the symptom-limited peak of incremental CPET.

### Sports bra conditions

Irrespective of bra condition, there was no significant difference in the bra underband pressure between SBV and LBV participants (*p* = 0.577, $\eta_{p}^{2}\;=\;0.02$), indicating that the two groups wore bras of similar tightness around the thorax. For the three bra conditions, the underband pressure of the low-support bra was greater than the high-support bra (57.8 ± 10.8 cmH_2_O vs. 44.4 ± 6.7 cmH_2_O, *p* < 0.001; *g* = 4.93) and the personal bras (37.2 ± 11.8 cmH_2_O, *p* < 0.001, *g* = 3.63), with no significant difference between high-support and personal bras (*p* = 0.058, *g* = 0.98). Average breast acceleration during the time-trial was 0.84 ± 0.73 cm/s^2^, with no significant difference across the three sports bra conditions (*p* = 0.650, $\eta_{p}^{2}\;=\;0.09$) or between SBV and LBV participants (*p* = 0.521, $\eta_{p}^{2}\;=\;0.08$).

### Time-trial performance

**Figure 2:**
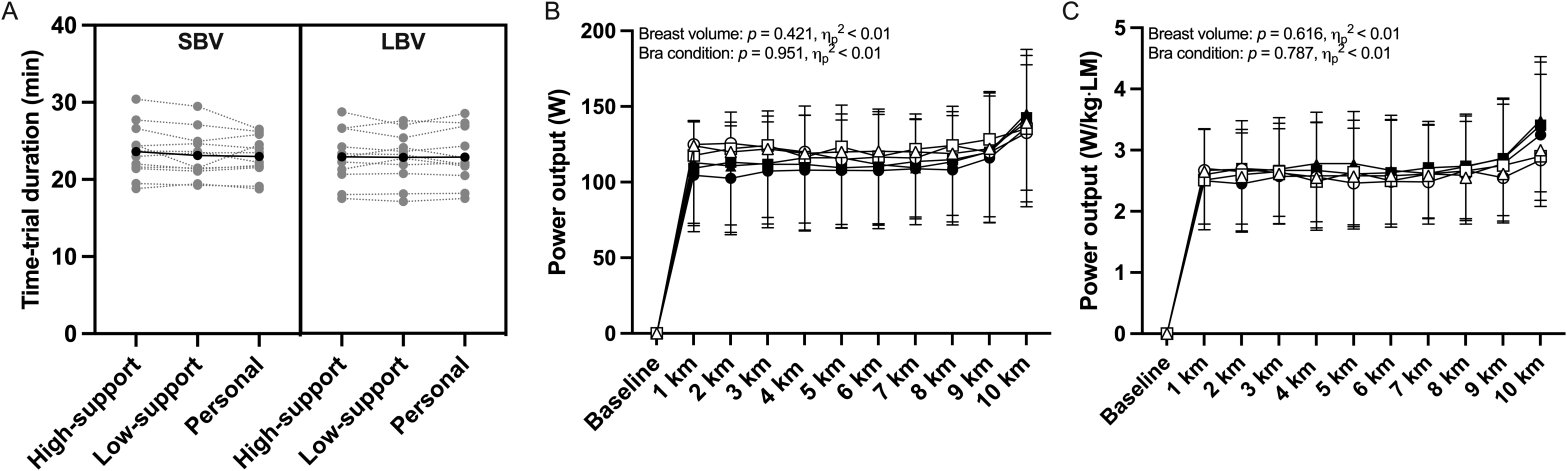
10-km cycling time-trial performance outcomes for small- and large-breast volume participants (SBV and LBV, respectively) while wearing a high-support, low-support, personal sports bras. **(A)** Individual data (grey markers) and group mean (black markers) for 10-km cycling time-trial duration. **(B**,**C)** Group mean data for absolute and relative power output during the time-trial. In panels B and C, SBV and LBV are represented by closed and open markers, respectively, while wearing high-support (●/○), low-support (▪/□), or personal sports bra (▴/▵). Bars and error bars represent mean ± SD for SBV (*n* = 12) and LBV participants (*n* = 11).

As shown in Figure 2A, the time-trial duration was 23.1 ± 3.1 min (range: 17.1–30.4 min), with no significant difference across the three sports bra conditions (*p* = 0.273, $\eta_{p}^{2}\;<\;0.01$) or between SBV and LBV groups (*p* = 0.794, $\eta_{p}^{2}\;<\;0.01$). The absence of a performance difference was further confirmed by no significant difference in absolute or relative power outputs between the two groups or bra conditions (see Figures 2B,C).

### Cardiorespiratory responses

**Figure 3:**
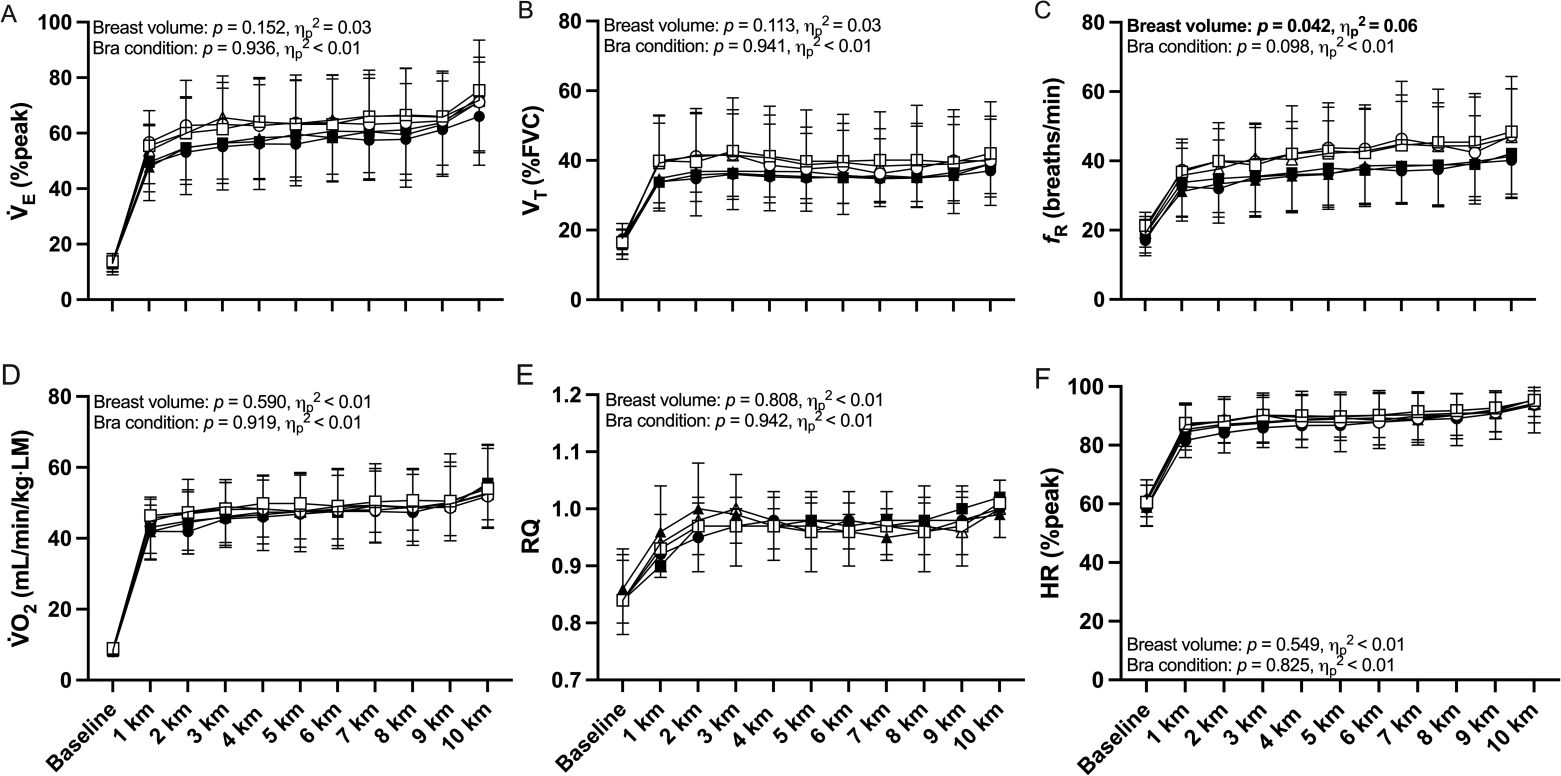
Minute ventilation expressed as a percentage of peak incremental minute ventilation (V̇_E_%peak), tidal volume expressed as a percentage of forced vital capacity (V_T_%FVC), respiratory frequency (*f*_R_), rate of oxygen uptake relative to whole-body lean mass (V̇O_2_ ml/min/kg·LM), respiratory quotient (RQ) and heart rate (HR) during 10-km cycling time-trial exercise in small-breast volume (SBV, *n* = 12, closed symbols) and large-breast volume participants (LBV, *n* = 11, open symbols) while wearing a high-support (●/○), low-support (▪/□), or personal sports bra (▴/▵), with associated *p*-values and effect sizes (partial eta squared, *η*_p_^2^) for main effects of sports bra and breast volume. Data are shown as mean ± SD.

There was no main effect of sports bra condition on any cardiorespiratory variable during the 10-km time-trial (see Figure 3). LBV participants maintained a significantly higher *f*_R_ (by ∼6 breaths/min) than SBV participants during the 10-km time-trial (small effect size, see Figure 3C). Otherwise, breast volume had no significant effect on any cardiorespiratory variable during exercise (all small effect sizes, see Figures 3D–F).

### Symptom responses

**Figure 4:**
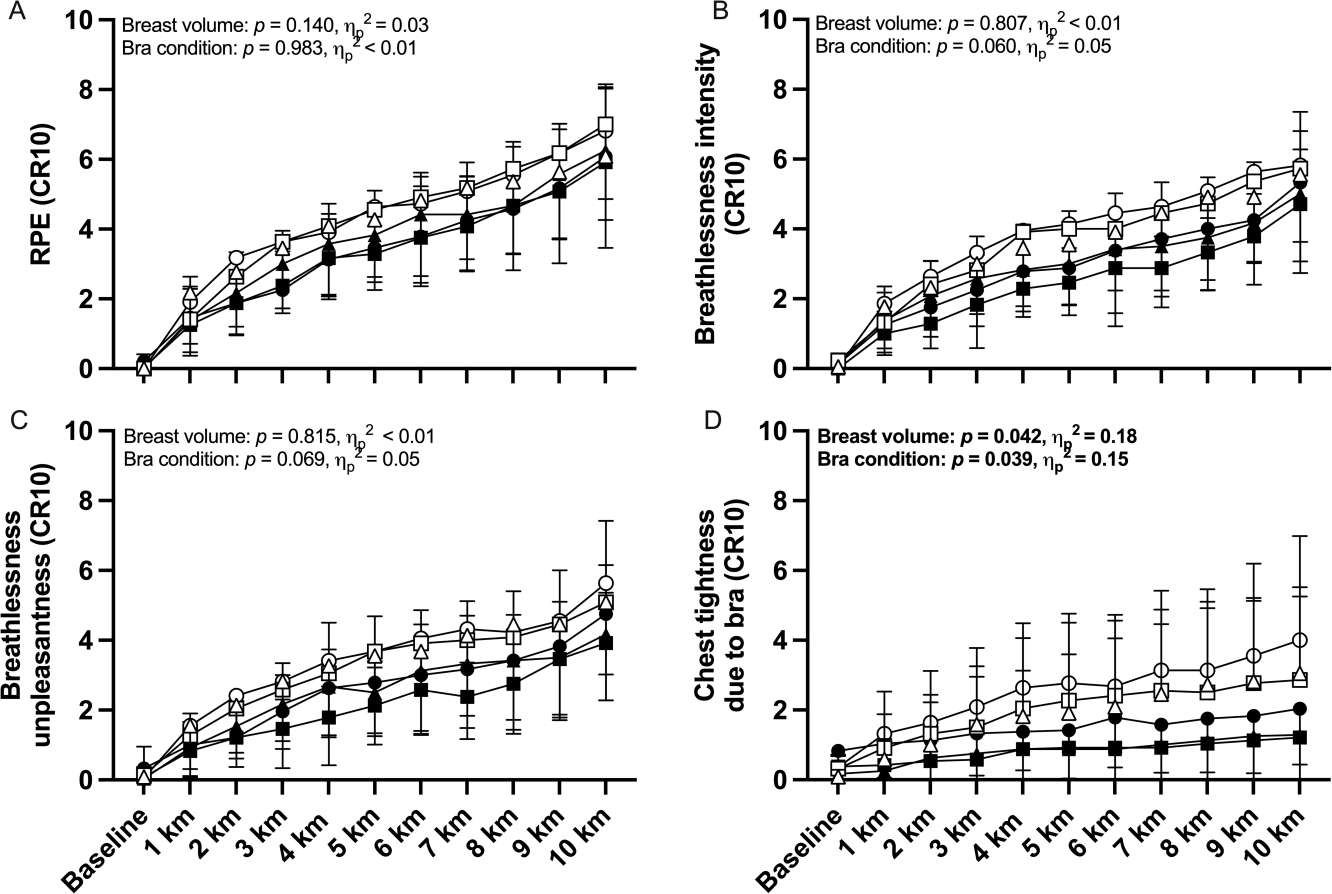
Modified Borg 0-10 category ratio scale (CR10) ratings of perceived exertion (RPE), breathlessness intensity, breathlessness unpleasantness, and chest tightness due to bra during 10-km cycling time-trial exercise in small-breast volume (SBV, *n* = 12, closed symbols) and large-breast volume participants (LBV, *n* = 11, open symbols) while wearing a high-support (●/○), low-support (▪/□), or personal sports bra (▴/▵), with associated *p*-values and effect sizes (partial eta squared, *η*_p_^2^) for main effects of sports bra and breast volume. Data are shown as mean ± SD.

There were no main effects of sports bra condition or breast volume on RPE, breathlessness intensity or breathlessness unpleasantness responses to exercise (Figure 4, all small effect sizes). There was, however, a main effect of both sports bra condition and breast volume on ratings of perceived chest tightness due to the bra (large effects, Figure 4D). Pairwise comparisons indicated that the high-support bra felt tighter and more restrictive around the chest than the low-support bra (*p* = 0.042, *g* = 0.56) but not the personal bra (*p* = 0.078, *g* = 0.62). Furthermore, pairwise comparisons for breast volume indicated that, compared to their SBV counterparts, LBV participants reported higher ratings of chest tightness due to their bras: 2.1 ± 1.2 vs. 1.0 ± 1.2 Borg 0–10 scale units (*p* = 0.046; *g* = 0.85). There was no interaction effect of bra condition × breast volume on ratings of perceived chest tightness due to bra (*p* = 0.947, $\eta_{p}^{2}$  < 0.01).

When correlating symptom responses from the two groups, the associations between ratings of perceived chest tightness due to the bra and each of breathlessness intensity, breathlessness unpleasantness, and RPE were nearly two-fold stronger in LBV vs. SBV participants (Table 3**,** all between-group comparisons *p* < 0.001).

**Table 3:** Correlation coefficients (*r*) of ratings of perceived exertion (RPE), breathlessness intensity, breathlessness unpleasantness, and chest tightness due to bra in small-breast volume (SBV) and large-breast volume (LBV) participants in upper and lower tables, respectively.

	RPE	Breathlessness intensity	Breathlessness unpleasantness
RPE	–		
Breathlessness intensity	0.881	–	
Breathlessness unpleasantness	0.756	0.886	–
Chest tightness due to bra	0.358	0.415	0.378
Data shown for SBV participants (*n* = 12).			
	RPE	Breathlessness intensity	Breathlessness unpleasantness
RPE	–		
Breathlessness intensity	0.953*	–	
Breathlessness unpleasantness	0.916*	0.946*	–
Chest tightness due to bra	0.696*	0.709*	0.711*

Data shown for LBV participants (*n* = 11). *p < 0.05 vs. SBV participants.

## Discussion

This is the first study to examine the individual and combined effects of sports bras and breast volume on 10-km cycling time-trial performance. First, we found no effects of sports bra condition or breast volume on exercise performance, i.e., 10-km cycling time-trial duration. Second, we found that the high-support sports bra was perceived as tighter around the thorax than the low-support and personal bras, but that exercise performance was nevertheless unaffected. Finally, we noted that LBV participants, irrespective of sports bra condition, experienced stronger symptoms of chest tightness due to their sports bras than SBV participants, and that the associations between ratings of perceived chest tightness, breathlessness (intensity and unpleasantness) and RPE were two-fold stronger in the LBV vs. SBV group. This latter observation may indicate that, while not detrimental to exercise performance in the context of this study, the awareness of bra-induced chest tightness may contribute to the overall subjective experience of self-paced exercise, especially in people with larger breast volumes.

### Sports bras and exercise performance

Bra-induced symptoms of chest tightness and rib cage restriction are well-established, both anecdotally and empirically (1, 3, 20). Sports bras—whether they are for recreational activities or competitive events—are intended to support breast tissue and prevent breast pain and discomfort resulting from excessive motion (21). However, the compressive characteristics of correctly-fitted sports bras may deter many from wearing these bras, with reports describing how the perceived chest tightness of a compression sports bra can cause premature exercise cessation in select individuals (1, 20). Others, again, may choose to exercise in bras that are not optimally fitted, thereby experiencing unnecessary breast pain, discomfort, and even embarrassment due to excessive breast movement (22).

Despite these reports, we have previously shown that bra-induced symptoms of chest tightness, rib cage restriction, and breathlessness (intensity and unpleasantness) are not manifestations of critical inspiratory constraints with greater inspiratory neural drive and respiratory muscle pressure generation during stationary bicycle exercise at low, moderate, or high intensities (3). In our earlier study, we also found that underband tightness (measured as the average of anterior and lateral underband pressure at end-inspiration, at which point the rib cage is expanded) was independent from bra-induced perceptions of chest tightness, as the sports bra that applied the greatest underband pressure (a low-support sports bralette) was perceived as the least restrictive to breathing. We thus continue to speculate that it is likely the unique designs of some sports bras (e.g., fabric choice, strap design, tightness over the cup, neckline, etc.), and not the underband *per se*, that evoke these stronger symptoms of chest tightness, rib cage restriction, and breathlessness.

After all, we found no difference in 10-km cycling time-trial exercise performance between the three sports bra conditions. Therefore, it is reasonable to assume that, despite experiencing greater chest tightness and rib cage restriction while exercising in the high-support sports bra, these symptoms were insufficient to make participants reduce their exercise intensity to an extent that impaired their performance. Indeed, the mean rating of chest tightness due to the bra during the final kilometer of the time-trials reached 2.9 ± 2.5 Borg units in the high-support sports bra vs. 2.1 ± 2.0 Borg units and 2.1 ± 2.1 Borg units in the low-support and personal bras, respectively. These values equate to descriptors of “slight (light)” to “moderate”, which were not sufficiently high to adversely affect performance in the context of this study.

### Implications for exercise performance

In line with our previous work (3), we hypothesized that LBV participants would self-select lower exercise intensities than SBV participants to avoid undue symptoms of chest tightness and breathlessness during the time-trials. Yet, our findings indicated that—despite a tendency for LBV participants to report stronger breathlessness and RPE throughout exercise than their SBV counterparts—these differences were not statistically significant and did not translate into performance differences.

We purposefully selected non-weight-bearing stationary bicycle exercise for this study to minimize the influence of breast motion, BMI, and body mass on our performance outcomes. We confirmed that the breast movement (acceleration) was negligible in all three sports bra conditions (mean ± SD 0.84 ± 0.73 cm/s^2^) relative to that reported during treadmill activities (23–25), confirming that we were able to isolate our physiological and symptom responses from breast movement and subsequent potential breast discomfort and/or pain. Under these controlled conditions, we found that there were no apparent differences between our sports bra conditions nor breast volume groups.

There have been no other attempts at studying the consequences of breast volume on non-weight-bearing cycle exercise performance. During marathon running, Brown & Scurr (26) estimated that—for females with bra underband sizes 32–38 (70–85 in European sizing)—each increase in cup size would equate to performance losses ranging from 4.6–8.6 min. In other words, a female with 36DD breast size would typically perform ∼34.4 min slower in the marathon than a comparable female with 36A breast size. While those authors did not adjust for BMI, training background, or previous marathon experience, the study was unique in estimating the potential implications of breast volume on prolonged weight-bearing exercise performance. Also during running, recent studies have found that females wearing low- compared to high-support sports bras unfavorably adapt their running technique to limit excessive breast motion, resulting in greater V̇O_2_ and poorer running economy (27, 28). Conversely, a recent study from Kipp et al. (2) indicated that an overly tight sports bra, as quantified by the pressure exerted by the underband on the thorax, may also have implications for whole-body metabolic rate. Specifically, those authors found that tightening the underband of a customized sports bra increased the total work of breathing by ∼6% alongside a concomitant increase in V̇_E_ (∼3%) and whole-body V̇O_2_ (by ∼1.3%) in highly trained female runners during submaximal treadmill exercise.

Although none of these studies compared SBV and LBV females, nor did they include a performance trial in their study protocols, their findings suggested that the adverse effects of a low-support sports bra on running mechanics, or even an overly restrictive sports bra on the respiratory system, can impair exercise performance via biomechanical and physiological effects on whole-body metabolic rate and, potentially, exercise performance.

### Methodological considerations

Our strategic choice of studying non-weight-bearing cycle exercise performance presents a limitation, as most people typically engage in weight-bearing activities like walking, hiking, jogging, and/or running. Transitioning from non-weight-bearing to weight-bearing exercise introduces a multifaceted challenge, where the metabolic cost of exercise increases (29) alongside increased breast motion and the potential for breast discomfort or pain (30–32). This places greater demands on sports bras, particularly in LBV females (33). So, while our study offers valuable insights within the context of non-weight-bearing exercise, its applicability to real-world scenarios involving weight-bearing activities necessitates careful consideration and additional research.

It is also pertinent to question whether our results extend to performances of longer durations. We chose the 10-km cycling time-trial (duration 17.1–30.4 min) for its high repeatability in recreationally-active individuals (17). The concept of “endurance” involves exercise lasting a few minutes to several days; each with unique physiological demands. Both our present and previous results (3) indicate that LBV females experience stronger symptoms of chest tightness compared to SBV females and that these sensations are strongly correlated with RPE and breathlessness (intensity and unpleasantness). In longer events, such as marathons, triathlons, and criterium races, it is reasonable to hypothesize that the prolonged experience of relatively greater exertional symptoms in LBV compared with SBV females wearing a professionally fitted sports bra could exert a more pronounced influence on emotion, self-selected exercise intensity and, consequently, overall performance. Additional research is needed to test this hypothesis and further explore the independent and combined effects of breast volume and sports bras on symptoms, physiology and biomechanics across a broader spectrum of exercise durations and intensities, providing a more comprehensive understanding of their interplay in diverse athletic contexts.

Finally, it is worth discussing how our study population may have affected our results. While we only included recreationally-active females for the purpose of generalization, it is possible that a study sample of endurance-trained athletes would have yielded different results. After all, an endurance-trained study sample would have been able to sustain higher relative work rates alongside higher levels of ventilation while also interpreting interoceptive cues (i.e., sensations of breathlessness, chest tightness, or fatigue) differently from a non-athletic sample (34).

## Conclusion

We showed no discernible differences in non-weight-bearing 10-km cycling time-trial exercise performance between SBV and LBV females, nor between optimally-fitted sports bras of various levels of support. We also showed that LBV females experienced stronger symptoms of chest tightness due to their sports bras compared to their SBV counterparts; sensations associated with greater RPE and breathlessness (intensity and unpleasantness) during exercise but nevertheless inconsequential for exercise performance under our experimental conditions. These findings contribute to understanding the complex inter-relationships between breast volume, sports bras and exercise performance in healthy, recreationally-active females. We urge that future research explore the broader implications of breast volume and sports bras across different exercise modalities, durations, and exercise intensities for females of different breast sizes in various athletic pursuits.

## Data Availability

The raw data supporting the conclusions of this article will be made available by the authors, without undue reservation.
