# Supplementary material for: Supported to perform: sports bras and breast volume do not impair cycling performance in females
Source: Front Sports Act Living. 2024 Oct 11;6:1439403. doi: 10.3389/fspor.2024.1439403 (PMC11502316; doi:10.3389/fspor.2024.1439403)
Supplement: Supplementary file 1 [file Image1.pdf]

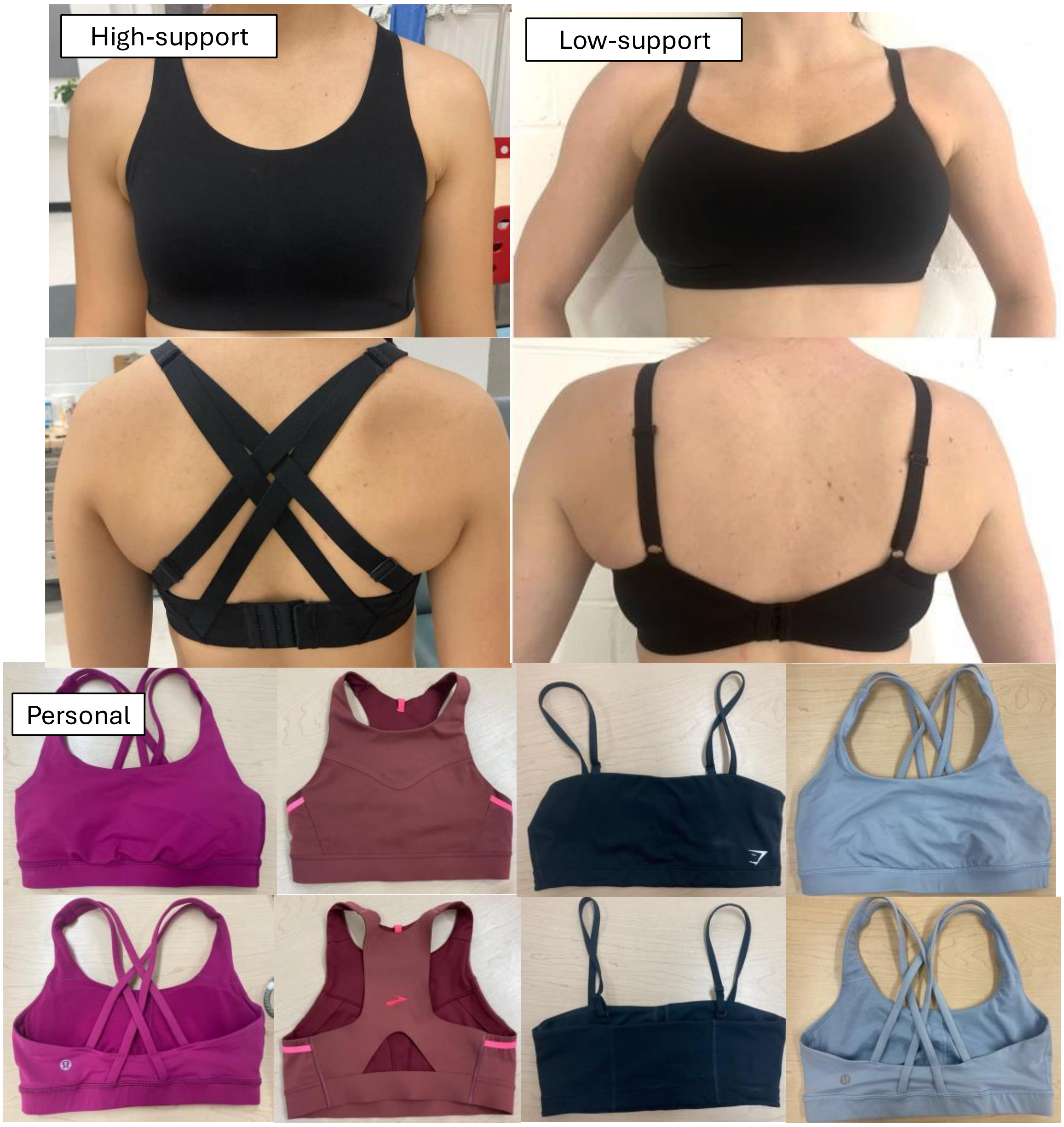

SUPPLEMENTAL FIGURE 1 Anterior and posterior views of experimental sports bras (high-support and low-support bras) and a selection of participants' personal sports bras for illustrative purposes.

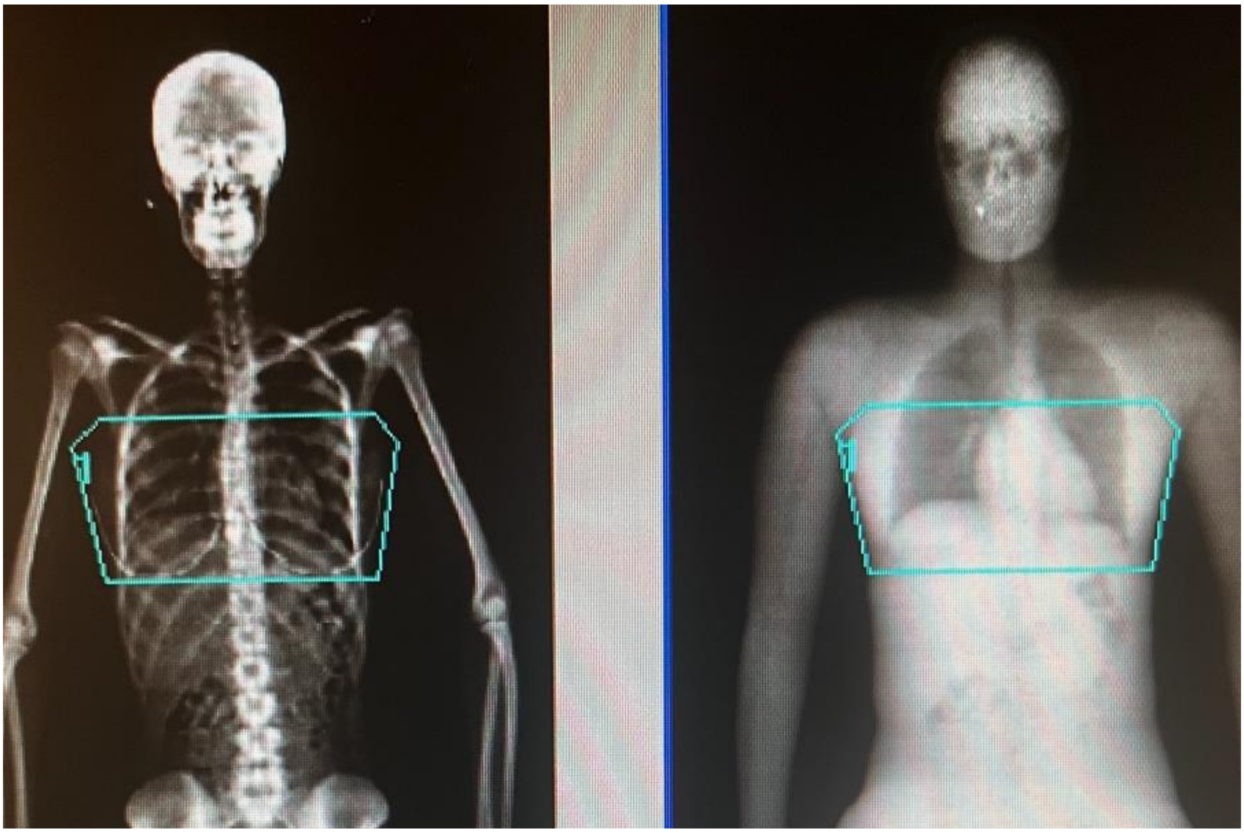

SUPPLEMENTAL FIGURE 2 To obtain measures of thoracic mass from dual x-ray absorptiometry (DXA) scans, a customized region of interest (ROI) was drawn as an elongated hexagon anchored between the axillae (armpits), vertically along the chest wall using the boundary between the skin and the dark background, down and horizontal across to the xiphoid process and the 6th rib. By using anatomical landmarks from the bone mineral density image (right) *and* soft tissue image (left), we could define our ROI along anatomical structures as well the tissue boundary at the chest wall. In some instances, we could also use the thin underwire of the participant's bra to guide our ROI (see Figure below). In LBV participants, it was particularly important to use the soft tissue image to guide our ROI, as the breast tissue was often extended beyond the rib cage when in a supine position. It is also worth noting that the same researcher (CI) processed and analyzed all DXA scans, thereby enhancing the reliability of these measures. As such, within-subject reliability was ensured by consistent positioning and scan protocols for each participant, minimizing variability in repeated measures. This approach helped to reduce measurement error and increase the accuracy of the outcomes.

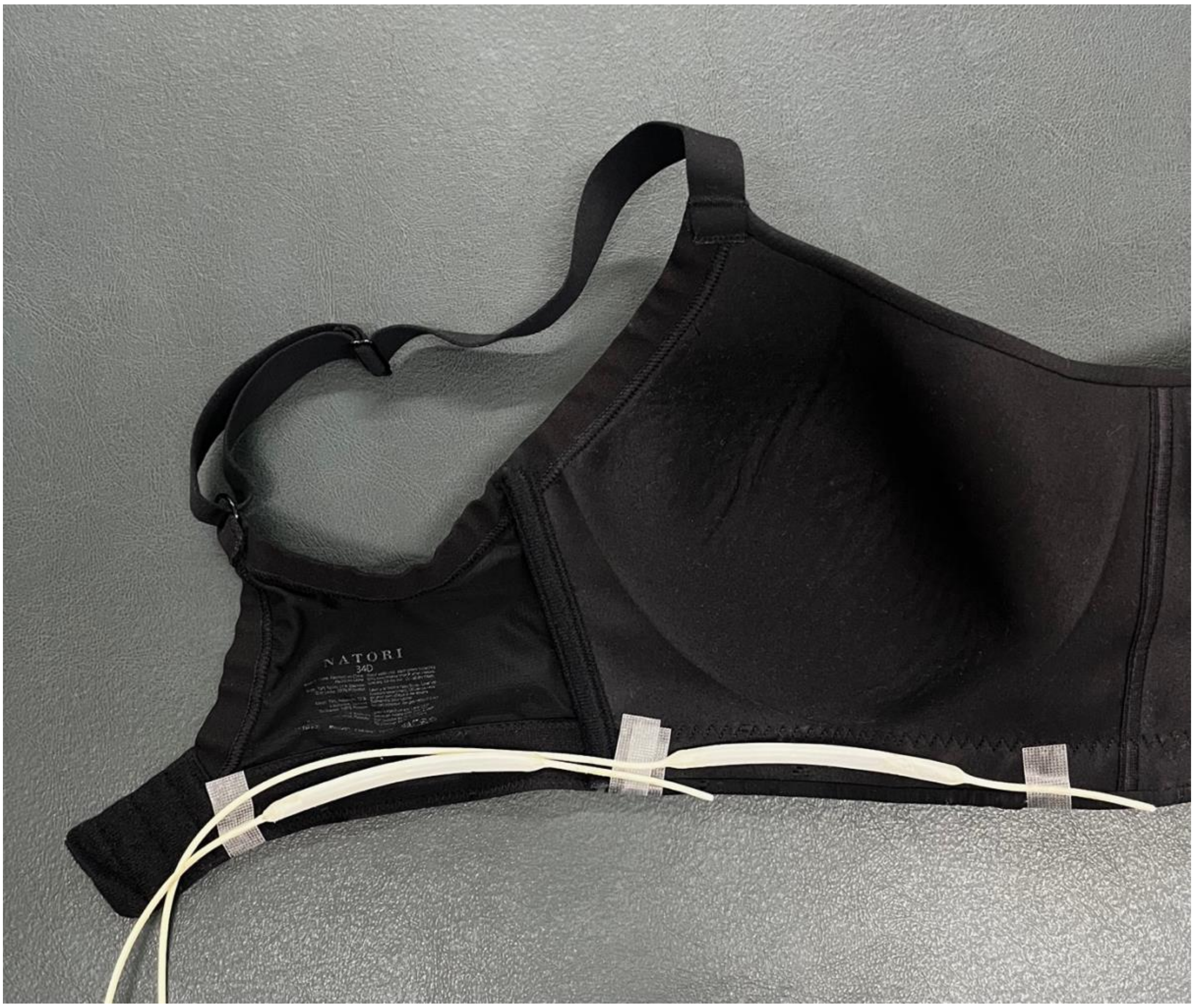

SUPPLEMENTAL FIGURE 3 The placement of balloon-tipped catheters underneath the underband of the sports bras. Both balloon-tipped catheters were taped on the non-inflatable parts and filled with 2 mL of air. The optimal balloon volume was identified in pilot work as the volume that did not collapse the balloon when applying the pressure of the underband against the thorax while not exceeding the balloons' working range.
